# Accuracy of height estimation and tidal volume setting using anthropometric formulas in an ICU Caucasian population

**DOI:** 10.1186/s13613-016-0154-4

**Published:** 2016-06-21

**Authors:** Erwan L’her, Jérôme Martin-Babau, François Lellouche

**Affiliations:** Réanimation Médicale, CHRU de Brest – La Cavale Blanche, Bvd Tanguy-Prigent, 29609 Brest Cedex, France; LATIM INSERM UMR 1101, Université de Bretagne Occidentale, Brest Cedex, France; Institut Universitaire de Cardiologie et de Pneumologie de Québec, Quebec, Canada

**Keywords:** ICU, Mechanical ventilation, Height estimation

## Abstract

**Background:**

Knowledge of patients’ height is essential for daily practice in the intensive care unit. However, actual height measurements are unavailable on a daily routine in the ICU and measured height in the supine position and/or visual estimates may lack consistency. Clinicians do need simple and rapid methods to estimate the patients’ height, especially in short height and/or obese patients. The objectives of the study were to evaluate several anthropometric formulas for height estimation on healthy volunteers and to test whether several of these estimates will help tidal volume setting in ICU patients.

**Methods:**

This was a prospective, observational study in a medical intensive care unit of a university hospital. During the first phase of the study, eight limb measurements were performed on 60 healthy volunteers and 18 height estimation formulas were tested. During the second phase, four height estimates were performed on 60 consecutive ICU patients under mechanical ventilation.

**Results:**

In the 60 healthy volunteers, actual height was well correlated with the gold standard, measured height in the erect position. Correlation was low between actual and calculated height, using the hand’s length and width, the index, or the foot equations. The Chumlea method and its simplified version, performed in the supine position, provided adequate estimates. In the 60 ICU patients, calculated height using the simplified Chumlea method was well correlated with measured height (*r* = 0.78; *∂* < 1 %). Ulna and tibia estimates also provided valuable estimates. All these height estimates allowed calculating IBW or PBW that were significantly different from the patients’ actual weight on admission. In most cases, tidal volume set according to these estimates was lower than what would have been set using the actual weight.

**Conclusion:**

When actual height is unavailable in ICU patients undergoing mechanical ventilation, alternative anthropometric methods to obtain patient’s height based on lower leg and on forearm measurements could be useful to facilitate the application of protective mechanical ventilation in a Caucasian ICU population. The simplified Chumlea method is easy to achieve in a bed-ridden patient and provides accurate height estimates, with a low bias.

**Electronic supplementary material:**

The online version of this article (doi:10.1186/s13613-016-0154-4) contains supplementary material, which is available to authorized users.

## Background

Knowledge of patients’ height is essential for daily practice in the intensive care unit (ICU), for either assessment of renal function [1], determination of drug doses, calculating cardiac function indices, or tidal volume setting [2]. Because it is well established that patients’ lungs are well correlated with their height [3], accurate tidal volume setting should be based on ideal or predicted body weight that is functions of height and gender, rather than on actual weight to avoid acquired acute lung injury and ARDS [4–6] and to improve outcome [7].

However, height measurement is not a daily routine in all ICUs [8–11]. Although recumbent patients’ height can be measured by means of a metric ribbon tape, this measurement is not always performed [12] or may lack consistency.

In fact, actual body weight is often used in routine [13], which can lead to large errors in tidal volume settings [14], especially in women and obese patients that are consistently at risk of unintentional delivery of excessive tidal volumes [7, 15–17]. Several other ICU team use height and weight estimates [9, 18, 19], but these visual estimations have yet been demonstrated as significantly inaccurate for individual observers [17, 20, 21].

In this study, we first analyzed on 60 healthy volunteers whether estimated height using various simple anthropometric formulas will agree with the exact measured height, and in second whether several formulas will help setting tidal volume in 60 mechanically ventilated ICU patients.

## Methods

This prospective observational protocol was in accordance with the standards of our local ethics committee; informed consent was not deemed necessary because of the observational nature of the study.

### Measurements and calculations

#### Height measurement

Exact height in the erect position (*actual height*) was considered the gold standard and was performed for all 60 healthy volunteers, using a standard clinical height gauge. However, such a measurement was unavailable for ICU patients and height measured with a soft tape metric ribbon in the supine position (*measured height*) was considered the standard for the 60 ICU patients; it was also measured for all healthy volunteers, as a comparison. Evaluation took also into account height provided on the healthy volunteers’ ID cards (*provided height;* unavailable for most ICU patients) and the visual estimation provided by the nurse in charge of the ICU patients (*evaluated height*).

#### Limb measurements and height estimations

**Fig. 1:**
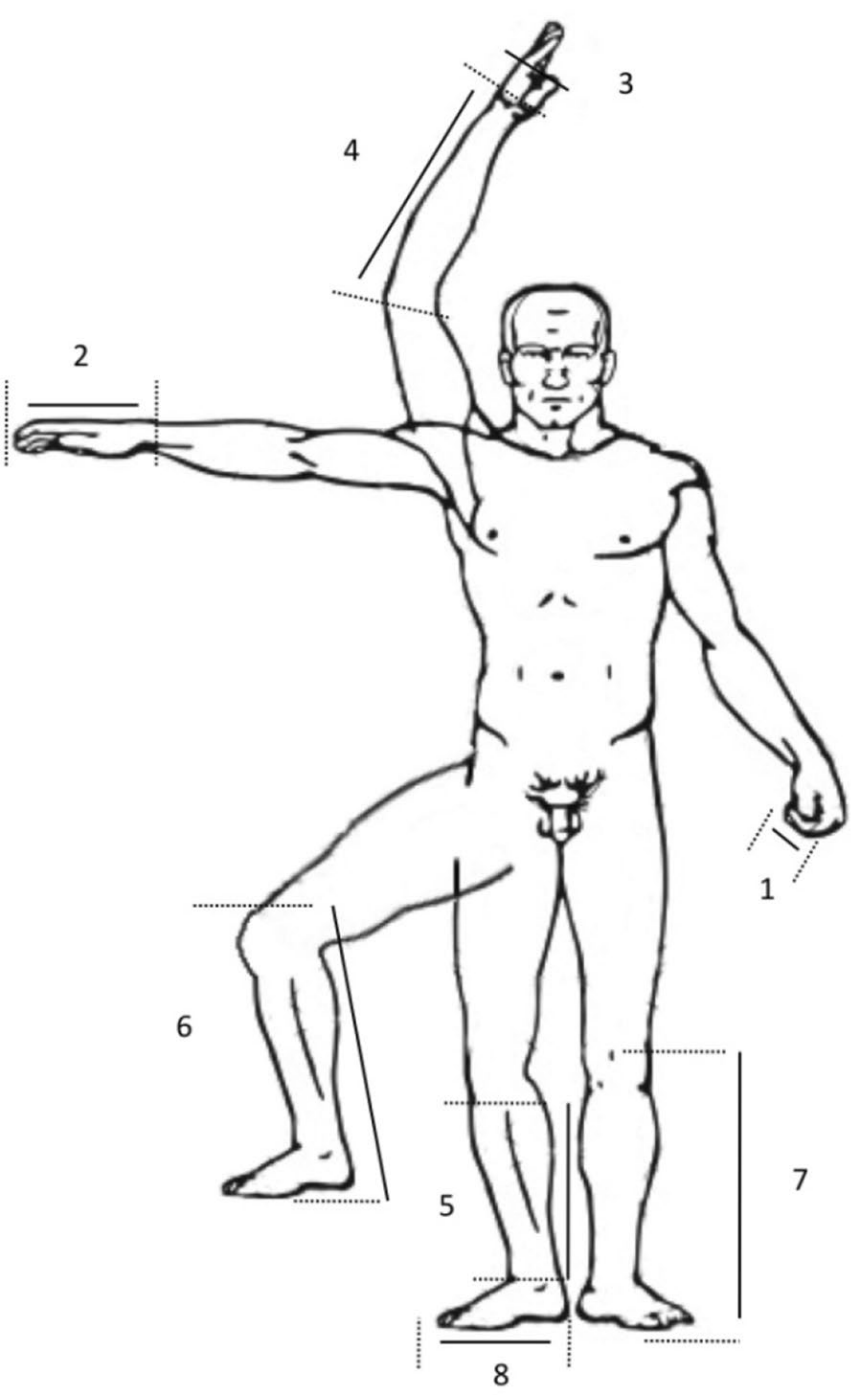
Limb segment measurements. All measurements were performed using precision callipers on the right limbs. *1* index distal phalange; *2* hand length, from the IIIrd finger extremity to the wrist; *3* hand maximal width; *4* ulna, from the olecranon to the styloïd process; *5* tibia length, from the upper articular line to the extremity of the medial malleolus; *6* standard Chumlea measurement, the patient is positioned recumbent, knee raised vertically with a 90° angle between femur and tibia, and the caliper is positioned under heel and over femoral condyle of the leg; *7* simplified Chumlea measurement, the patient stays supine and the caliper is positioned under heel and over patella’s upper line; *8*: foot length, from the extremity of the Ist toe to the posterior part of the heel

They were performed using 300- and 800-mm precision metal callipers. During the preliminary phase, on healthy volunteers, eight different limb measurements (Fig. 1) were performed, always on the right limbs, to determinate height estimation (*calculated height*) using different anthropometric formulas (Additional File 1) [22–29].

#### Weight measurement and calculation for ICU patients

All patients were weighted on admission using their ICU bed integrated weight scale (Total Care^®^ P500, Hill-Rom, Batesville, IN, USA). Ideal or predicted body weight was calculated using the different height values [30, 31].

A specific computer software application was designed to facilitate height evaluation during the second phase of the project. The choice of four equations (up from 18 used in the preliminary phase) used to evaluate height in the application took into account either accuracy and/or practical issues about the limb sections measurements.

### Preliminary phase on healthy volunteers

Height and limb segment measurements were performed over a 60 healthy volunteers’ cohort: four at the upper limb and four at the lower limb (Fig. 1). Height estimates are provided in Table 1.

**Table 1:** Height measurements and estimations in the healthy volunteers

Healthy volunteers (*n* = 59)	Measure (cm)	Correlation (*r*)	Bias (%) (±1.96 SD)
Actual height	*170.4* *±* *8.5*	/	/
Provided height	170.2 ± 8.1	0.9633	−0.1 (2.4/−2.6)
Measured height	176.2 ± 8.5	0.9795	3.4 (5.3/1.4)
Height estimations			
Index			
I 1	164.3 ± 7.2	0.7141	−3.6 (3.4/−10.7)
I 2	167.3 ± 5.4	0.7300	−1.7 (5.0/−8.5)
Hand length			
HL 1	163.2 ± 7.7	0.7915	−4.3 (1.9/−10.4)
HL 2	160.9 ± 7.2	0.7938	−5.7 (0.3/−11.7)
HL 3	161.4 ± 5.6	0.7646	−5.3 (1.0/−11.7)
Hand width			
HW 1	155.8 ± 6.8	0.7903	−8.9 (−2.8/−14.9)
HW 2	157.7 ± 6.6	0.6962	−7.9 (−0.9/−15.0)
Ulna			
U 1	169.7 ± 8.0	0.8296	−0.4 (5.2/−5.9)
U 2	172.2 ± 8.0	0.8296	1.1 (6.6/−4.4)
U 3	171.5 ± 9.4	0.7961	0.6 (7.2/−6.0)
Tibia			
T 1	162.1 ± 6.2	0.7989	−4.9 (0.9/−10.8)
T 2	158.8 ± 8.8	0.8443	−7.1 (−1.3/−12.8)
T 3	165.6 ± 9.2	0.8501	−2.9 (2.8/−8.5)
T 4	168.1 ± 9.2	0.8501	−1.4 (4.2/−6.9)
T 5	164.7 ± 10.7	0.8287	−3.5 (3.5/−10.4)
Foot	166.2 ± 8.6	0.0269	−2.6 (11.6/−16.8)
Chumlea			
Reference Chumlea	168.9 ± 6.0	0.7894	−0.8 (5.3/−6.9)
Simplified Chumlea	168.1 ± 5.9	0.8667	−1.3 (3.9/−6.4)

Correlation coefficient r and bias: results are provided as compared with actual height; measures are provided as mean ± SD

Actual height: height measured in the erect position using a vertical calliper; provided height: height provided by the healthy volunteers; measured height: height measured in the supine position, using a soft tape metric ribbon

Index: two different formulas were used (I 1, 2); hand: five formulas were used, three based on length (HL 1–3) and two combining width and length (HW 1, 2); ulna: three formulas were tested (U 1–3); tibia: five formulas were tested (T 1–5); reference Chumlea was measured with the patient positioned recumbent, knee raised vertically with a 90° angle between femur and tibia, and the calliper positioned under heel and over femoral condyle of the leg; simplified Chumlea was measured with the patient laying supine and the calliper positioned under heel and over patella’s upper line (see Fig. 1). All formulas are provided within the Additional file 1: Online Repository

Italic value indicates the reference value for height, that was measured in the erect position

Using these measurements, *calculated height* was performed using 18 different anthropometric formulas (Table 1). Complete anthropometric formulas are provided within the Additional file 1: online repository. They were chosen either because of their standard use within different domains such as geriatrics, anthropometry, and/or forensic science or because of pragmatic issues. Only a few of them are specifically dedicated to a European Caucasian population. The simplified Chumlea method was proposed by our team after several preliminary tests (*data not shown*), using the same equation but performing different measurements to better fit to ICU requirements (Fig. 1).

The four most accurate estimation indices were subsequently chosen (two for each limb segment), taking into account their accuracy (a correlation >0.75 and bias level <5 % as compared with *actual height* were considered), but also practical issues such as the measurements ease in the recumbent position; all chosen measurements had to be performed in a “one shot,” using a single 1-m ruler, as a comparison with the actual height measurement that requires several steps to be performed. These four indices were integrated into the specifically dedicated software application.

### Second phase on ICU patients

After the preliminary phase, weight, limb segment, and height measurements were performed in 60 consecutive mechanically ventilated ICU patients, for whom preadmission values were unknown to staff. Each patient’s height was estimated by eye while the patient was lying supine. Tidal volume (Vt) and plateau pressure were recorded concomitantly.

Height and predicted body weight (PBW) estimations [30] were performed retrospectively, and no direct intervention was immediately driven taking into account these evaluations. Ideal body weight (IBW) [31] was also computed for the sake of comparison to PBW.

### Statistical analysis

Anthropometric formulas have already been validated on various cohorts; however, few of them have been validated for a clinical use in our population of interest, except for the original Chumlea index [29]. For such a preliminary evaluation, a number of 60 healthy volunteers and 60 mechanically ventilated patients for more than 48 h were determined a priori.

All results are provided as mean ± SD, unless specified otherwise. Categorical variables are presented as counts. Relationship between variables was assessed using a Pearson’s correlation coefficient (*r*) test with p value, and data were represented graphically by a scatter diagram depicting the identity line and the 95 % confidence interval (CI) for *r*. Method comparison and evaluation was performed using a Bland–Altman plot, taking into account the difference between the two methods on the X-axis, because all comparisons were performed considering the reference method [32]; bias (*∂*) plot either was reported using quantitative differences or expressed as % of difference, depending on the value type. Quantitative parameter comparisons were made using paired *t* test. A p value equal or below 0.05 was considered statistically significant.

All statistical analyses were performed using MedCalc® 12.4.0 software for Windows (MedCalc Inc., Ostend, Belgium).

## Results

### Healthy volunteers

In the 60 healthy volunteers, *actual height* was well correlated with *provided* and with *measured* heights, with a low estimation bias (*∂* = −0.1 and 3.4 %, respectively).

#### Upper limb equations

Correlation was low between actual and calculated height, using either the hand’s length and width, or the index equations. Correlation between *actual height* and *calculatedheight* using the *ulna* was considered of interest, with a low bias (*∂* = −0.4–1.1 %).

#### Lower limb equations

Correlation was considered of interest whatever the tibia formulas, but with differences in terms of the estimation bias. Foot estimation was not correlated with *actual height* in our population. Height estimation using either the reference Chumlea method or the simplified one seemed to provide adequate values.

#### Choice of the anthropometric formulas for the second phase, within the ICU environment

 Considering either the performance of the different equations or the ease of measurements at the bedside, we chose to consider U1 (ulna) and HL1 (hand length) formulas for the upper limb, T4 (tibia) and SC (simplified Chumlea) for the lower limb.

### ICU patients

Patients’ physiological characteristics are provided in Table 2.

**Table 2:** ICU patients’ physiological characteristics

	ICU patients (*n* = 59)	*P* value
Diagnosis		
Cardiac arrest	18	
Coma	14	
ARDS	11	
Severe sepsis and shock	10	
ARF	4	
Other	2	
Patients’ characteristics		
Sex ratio	41 male/18 female	
Actual body weight (ABW; kg)	74.4 ± 16.2	
Ideal body weight (IBW; kg)	64.1 ± 6.5	*P* < 0.0001
Predicted body weight (PBW; kg)	64.1 ± 8.8	*P* < 0.0001
Ventilatory settings		
Respiratory rate (b/min)	18 ± 3	
Plateau pressure (cmH_2_O)	19 ± 4.5	
Vt (mL)	500 ± 56	
Vt ABW (mL/kg)	7.0 ± 1.4	
Vt IBW (mL/kg)	7.8 ± 0.8	*P* < 0.0001
Vt PBW (mL/kg)	7.9 ± 1.0	*P* < 0.0001

Results are provided as mean ± STD. A *p* value equal or below 0.05 was considered statistically significant

*ARDS* acute respiratory distress syndrome, *ARF* acute respiratory failure, *Vt* tidal volume, *ABW* actual body weight, *IBW* ideal body weight, calculated according to the Lorentz formula (ref), *PBW* predicted body weight; both reference IBW and PBW were calculated using measured height. IBW and PBW were significantly different from ABW, but without difference between each other; Vt ABW is the tidal volume that was set on the ventilator, according to the patient actual weight, measured on admission; it was significantly different from either Vt IBW or Vt PBW, without any difference between each other

Evaluated height (visual estimation) was correlated with the measured value (metric ribbon tape), with a low bias (*∂* < 1 %). Calculated height using the simplified Chumlea method was well correlated with measured height (*r* = 0.78; *∂* < 1 %) (Table 3).

**Table 3:** ICU patients’ height and weight estimations and calculated tidal volumes

	Measure (cm)	Correlation (*r*)	95 % CI for *r*	Bias (%) (±1.96 SD)
Measured height	169.5 ± 8.1	/	/	/
Estimated height	170.2 ± 8.1	0.77	0.64–0.86	0.4 (6.8/−6)
Height estimations				
Hand	163.3 ± 7.4	0.53	0.32–0.70	−3.8 (5.0/−12.7)
Ulna	165.2 ± 7.2	0.51	0.29–0.68	−2.6 (6.4/−11.5)
Tibia	174.2 ± 7.6	0.61	0.41–0.75	2.7 (10.9/−5.5)
Simplified Chumlea	162.2 ± 9.0	0.78	0.66–0.87	−4.5 (2.5/−11.5)
	*Weight (kg)*			
PBW estimations	(64.1 ± 8.8)			
Hand	58.5 ± 8.6	0.65	0.46–0.77	−10 (12.7/−32.8)
Ulna	60.2 ± 7.8	0.64	0.45–0.77	−6.6 (14.3/−27.5)
Tibia	68.4 ± 7.9	0.71	0.55–0.82	6.2 (24/−11.6)
Simplified Chumlea	57.5 ± 9.9	0.81	0.70–0.88	−12.2 (12/−36.4)
IBW estimations	(64.1 ± 6.5)			
Hand	59.7 ± 5.7	0.62	0.43–0.76	−7.2 (9.7/−24)
Ulna	60.9 ± 5.5	0.61	0.42-0.75	−5.0 (11.8/−21.8)
Tibia	67.2 ± 6.0	0.71	0.55-0.82	4.7 (19.8/−10.4)
Simplified Chumlea	59.0 ± 6.6	0.81	0.70–0.89	−8.5 (4.7/−21.6)
	*Tidal volume (mL/kg)*			
Vt over ABW	(7.0 ± 1.4)	/	/	/
VT over PBW	(7.9 ± 1.0)	/	/	/
Hand	8.7 ± 1.3	0.67	0.50–0.79	7.2 (24.1/−9.6)
Ulna	8.4 ± 1.3	0.69	0.53–0.81	5.0 (21.8/−11.8)
Tibia	7.4 ± 1.0	0.66	0.48–0.78	−4.7 (10.4/−19.8)
Simplified Chumlea	8.9 ± 1.7	0.84	0.74–0.90	8.5 (21.6/−4.7)
VT over IBW	(7.8 ± 0.8)	/	/	/
Hand	8.4 ± 1.0	0.71	0.55–0.82	9.6 (32.2/−13)
Ulna	8.2 ± 1.0	0.75	0.61–0.84	6.2 (28.8/−16.4)
Tibia	7.5 ± 0.8	0.74	0.60–0.84	−6.6 (13.9/−27.1)
Simplified Chumlea	8.5 ± 1.1	0.85	0.76–0.91	11.8 (31.4/−7.8)

Results are provided as mean + STD; correlation coefficient *r* and bias: results are provided as compared with measured height; ideal body weight is calculated according to the Lorentz formula, using the measured height; predicted body weight is calculated according to the ARDSnet tables, using the measured height

A significant difference was observed between actual body weight (ABW), measured on ICU admittance, and either IBW or PBW. In all cases, IBW and PBW were lower than ABW.

IBW and/or PBW calculations using the height calculated values were well correlated with values provided using the measured height.

Tidal volume on admission was significantly higher than that suggested while using IBW and/or PBW. In all cases, tidal volume settings using calculated height (whatever the chosen formula) were below those using ABW.

## Discussion

Because actual height may be difficult to obtain in all bed-ridden ICU patients, we compared different alternative methods to estimate height in 60 healthy volunteers and validated its usability in 60 ICU patients. Several alternative calculating methods, based on lower and upper limbs measurements, were close to the reference. When used for ventilation setting, such alternative, simple, and accurate height estimations mostly tended to decrease calculated predicted body weight, thus decreasing the risk of high tidal volume administration.

### Lack of accurate height measurements in ICU patients

Despite the paucity of data, several studies suggest that height is not routinely used to set tidal volume [14, 18, 33–35] and/or that the exact patient’s height is unknown as up to 40 % in ARDS patients [36]. In a UK telephone survey performed in 20 ICUs, the authors demonstrated that only 2 ICUs were using actual height for tidal volume setting [9].

When height measurements are performed, metric ribbon tape measurements are used in supine patients, even if it has proved to lack consistency in various studies [17, 20]. Such measurements have also been demonstrated to result in different height values than that would be obtained with the patient in the upright position [9]. A reason for such low performance could be that measurement is difficult to achieve by a single operator on a bed-ridden patient, especially in case of body distortion, obesity, and other physiological conditions.

In other studies, visual height estimation was the only method to be used, even if it seemed to be usually inconsistent [17, 21]. The magnitude of errors for visual estimation of height in the ICU varies from one study to the other, but several authors have depicted <41 % accuracy [20]. Most of all, experience and the level of training did not correlate well with accuracy of the estimations [17]. Such bad performance of clinicians to visually estimate physiological parameters for patients lying supine was also clearly demonstrated in the operating room, for either adults [37] or pediatrics [38], and in the emergency department [39]. In the operating room studies, marked variations were demonstrated between different observers for a single patient [37, 38].

Similar height misestimating was not observed in our study, as within a nursing study from the Netherlands [12], and measurement in supine or upright positions was well correlated in healthy volunteers. The accuracy of height measurement in such setting may be related to the fact that (1) in an experimental setting, we always try to provide the most accurate measurement, which may not always be the case in daily ICU routine measurements; (2) physical condition of healthy volunteers may have simplified measurements (no distortion, no obesity, etc.).

### Alternative methods for height estimation

Numerous methods have been described to calculate patients’ height indirectly, most of them being developed for anthropologic or forensic purposes. These methods used either a multiple regression approach with different bones measurements or a simple regression logistic [28]. Only a few of these simple methods have been developed for a clinical purpose and rarely in a European and Caucasian population [26].

Besides these methods, the long bone length is often considered the best indicator of stature, and knee height has been validated for stature evaluation using the Chumlea method [29] in large cohorts of mobility-impaired and bed-ridden elderly patients, close to a standard ICU population [40]. Despite promising results, knee cannot easily be raised vertically with a 90° angle between femur and tibia as in the standard method on a clinical routine [41]—especially in case of femoral venous access and/or overweight. Within all the other height estimation methods, few can yet be considered as reliable for a clinical purpose [42].

**Fig. 2:**
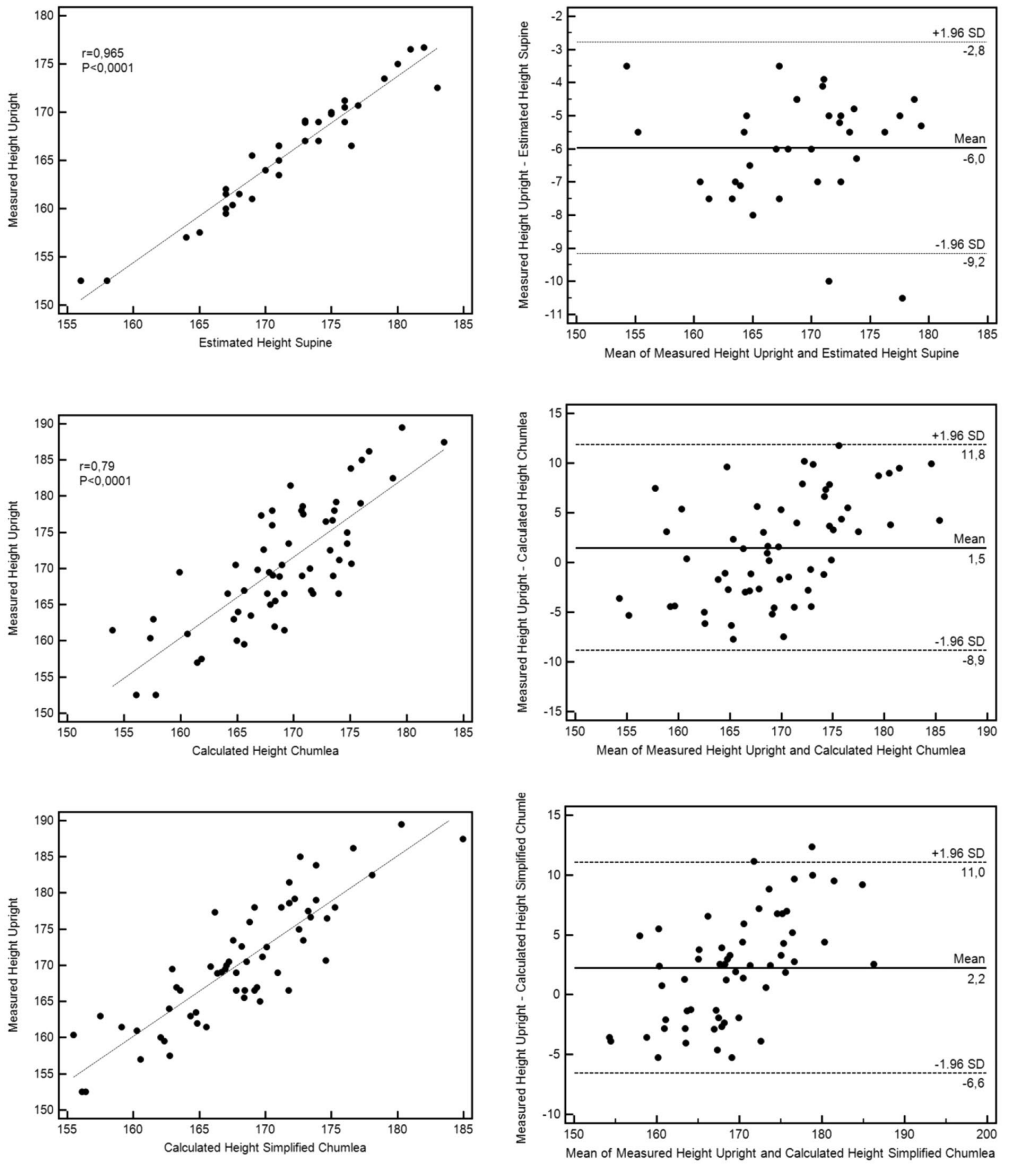
Comparison of different methods for height evaluation in healthy volunteers. The *left column* represents the regression diagram of the two tested methods. The independent variable (reference value = measured height in the erect position) defines the *vertical axis*, and the dependent variable (tested method) defines the *horizontal axis*. *Dark line* represents the regression line; *r* = correlation coefficient; *P* value ≤0.05 was considered significant. The *right column* displays the scatter diagram of the differences of the two methods (Bland and Altman plot). *Dark line* represents the mean difference (estimation bias = *∂*) between the two methods; *dotted line* represents the limit of agreement (plus and minus 1.96 SD) of the differences. For healthy volunteers, measured height in the upright position (reference) was well correlated with measured height in the supine position. This measured height may induce errors of 9.2 cm (2/60 volunteers with an error >10 cm). Chumlea height estimation, using either the standard or the simplified method in the supine position, was well correlated with actual height, with a low estimation bias. It may, however, induce errors from 8.9 to 11.8 cm

The simplified Chumlea method that is described in this article does not require such leg mobilization and can be easily achieved in supine patients by a single clinician, only using a short disposable ribbon tape, whatever the patient’s morphology. It seems to provide valuable height estimation, similar to what has been demonstrated with the original version. A relationship seems to exist between actual height and the two different Chumlea estimates (Fig. 2); i.e., the difference is depending on the height (overestimation of height for higher individuals).

### Potential impact of height calculation on protective ventilation implementation

Tidal volume is directly related to the exact patient height [3], and the absence of height value reference may lead to large errors in tidal volume setting [14]. The association between initial high tidal volume settings and acute lung injury or ARDS development has been clearly demonstrated [5, 6].

In numerous studies, obese patients were considered to be ventilated with higher tidal volumes than non-obese patients [7, 15–17]. Women of shorter height are thus less likely to receive protective ventilation [43, 44]. These detrimental effects could be directly related to the fact that these categories of patients may be ventilated using actual body weight or bad estimates [13].

**Fig. 3:**
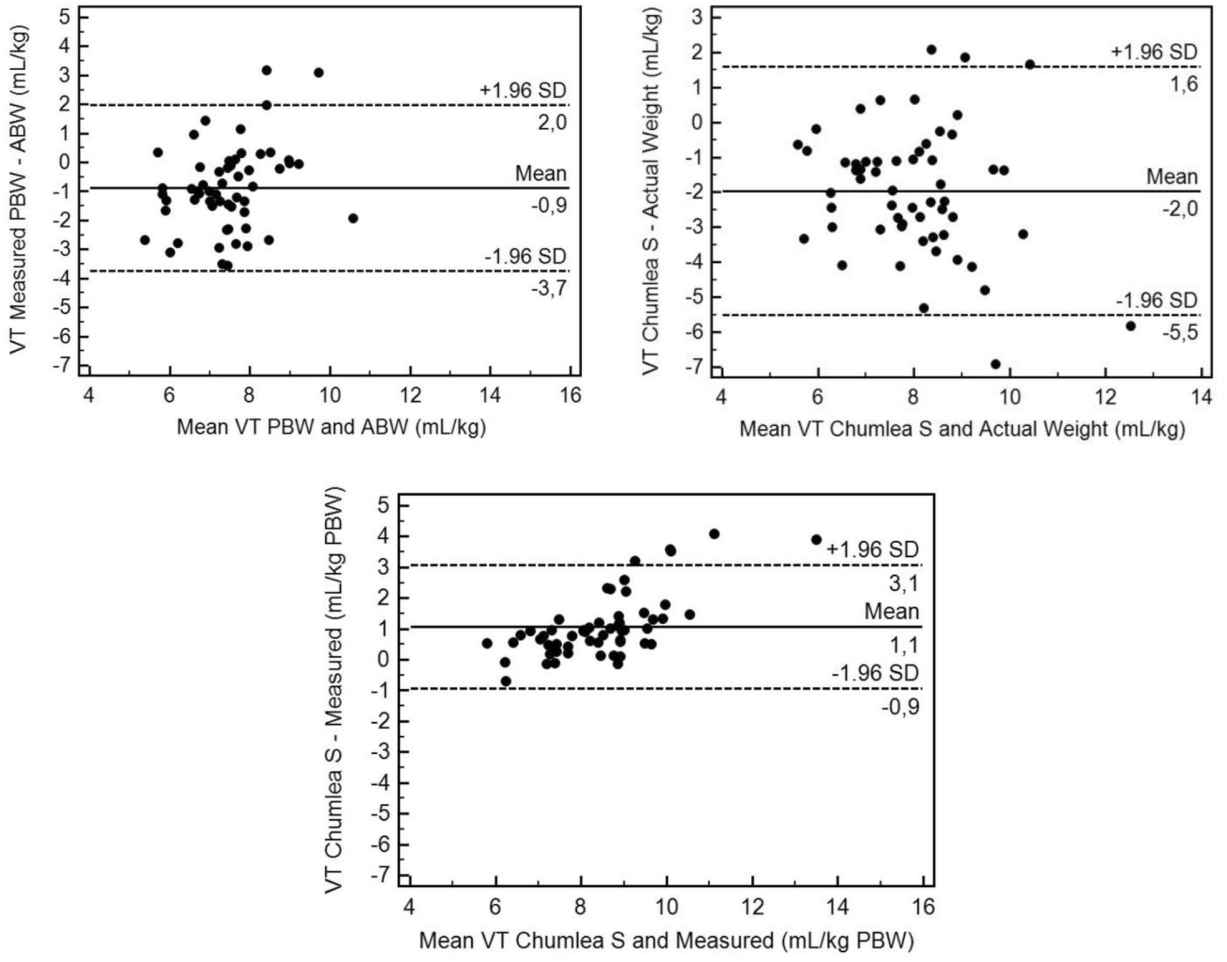
Bland and Altman plot for tidal volume in ICU patients, using various measures and estimates. *VT* tidal volume, *PBW* predicted body weight, *ABW* actual body weight, *VT Measured* tidal volume set using the measured height, *VT Chumlea S* tidal volume set using the Simplified Chumlea height estimate. Tidal volume setting grandly vary while using either PBW or ABW, with as much as a 3.7 mL/kg range, whereas the mean bias remains low (−0.9 mL/kg). VT settings using either the measured PBW or its estimate (visual height estimation) are consistent. The simplified Chumlea method is consistent with the one using the measured value, generally providing a 1.1 mL/kg lower value

In some of our patients, although very few obese patients were included, 6 mL/kg of actual body weight value would be the equivalent of 10–11 mL/kg of the ARDSnet approach. In the report by Bloomfield et al. [17], using 6 mL/kg of actual body weight in some patients may have resulted in tidal volumes of 15–19 mL/kg of the ARDSnet approach (Fig. 3).

Whatever the calculation formulas that are used, tidal volume settings errors are limited, whereas height estimates are usually higher than exact height. This error also tends to limit tidal volume/kg application. While the error in terms of calculation seems to be depending on the height, for patients over 170 cm, this will always lower the estimation of required tidal volume. The clinical impact of such an approach should require a dedicated study, but the availability of height estimates that are simple, easy to use, and rapid to perform will at least enable the clinician to titrate tidal volume as safely as possible with sufficient accuracy.

A note of caution could be that if the rationale of the study is supported by RCTs showing the benefits of tidal volume reduction based on PBW, height estimates were not similarly performed within these trials, thus probably resulting in some inconsistency. The question that we addressed could make sense from a clinical point of view, whereas our technique could help standardizing height estimation using a simple, cheap, and reproducible technique.

### Limitations of the study

Our study has several major limitations. The first limitation to consider should be the lack of exact height measurement for ICU bed-ridden patients. Even if metric ribbon measurements in the supine position cannot be considered as accurate as to height measurement in the erect position, it is often the only available reference for bed-ridden patients. As a matter of fact, this was the only comparable measurement that was available in our ICU survey. Second limitation could be that although height was not measured before study entry, it is unknown whether the nursing staff that was asked for visual estimation and/or metric ribbon measurements had prior knowledge of the patient’s height from other sources such as the patient’s family, the patient itself (rarely available at ICU admittance), or the patient’s medical record and/or ID. This may have artificially enhanced the exactitude of visual estimation. Patients’ position in a bed of already known length may also have bias estimation by expert nurses. Third limitation could be that Chumlea stature prediction equations have been made specifically for defined populations [26, 29] and that other alternative methods have been developed and should be used for differing populations [45, 46]. However, such a limitation has been emphasized within the first phase of the study that was dedicated to the choice of the most accurate estimation formulas within our population of interest. Fourth limitation should be the fact that regression formula validation requires a huge cohort of patients, which is not the case within the current study. However, one should also consider that all the formulas for height estimates that were used within the study have already been validated and that the study only applies such formulas in a different setting. Fifth and last limitation could be that our reference weight was measured on admission, while some of the patients may already have received huge amounts of intravenous fluids.

## Conclusion

When actual height is unavailable in ICU patients, alternative anthropometric methods based on lower leg and forearm measurements could be useful to calculate patient’s height and to facilitate the application of protective mechanical ventilation. The simplified Chumlea method is easy to achieve in a bed-ridden patient and provides accurate height estimates, with a low bias. Ulna and tibia estimates also provided valuable height estimates. All these methods are easy to perform, probably less time-consuming than standard methods, and they can also be performed with a short-length disposable tape instead of using long-lenght reusable tape, in an attempt to limit cross-contamination.

## Additional file

10.1186/s13613-016-0154-4 The seventeen different height estimation formulas that were used for the different measures are presented. Ten estimation formulas are proposed for the upper limb and seven for the lower limb; the modified Chumlea method uses different marks, but the same calculation formula.

## Abbreviations

ICU: intensive care unit
ARDS: acute respiratory distress syndrome
ID: identification card
ABW: actual body weight
IBW: ideal body weight
PBW: predicted body weight
CI: confidence interval
*r*: Pearson’s correlation coefficient
*∂*: bias
I 1, 2: different index measurements
HL 1–3: different hand length measurements
HW 1, 2: different hand width measurements
U 1–3: different ulna measurements
T 1–5: different tibia measurements
ARF: acute respiratory failure
Vt: tidal volume
