# Supplementary material for: Accuracy of height estimation and tidal volume setting using anthropometric formulas in an ICU Caucasian population
Source: Ann Intensive Care. 2016 Jun 21;6:55. doi: 10.1186/s13613-016-0154-4 (PMC4916127; doi:10.1186/s13613-016-0154-4)
Supplement: Supplementary file 1 — 10.1186/s13613-016-0154-4 The seventeen different height estimation formulas that were used for the different measures are presented. Ten estimation formulas are proposed for the upper limb and seven for the lower limb; the modified Chumlea method uses different marks, but the same calculation formula. [file 13613_2016_154_MOESM1_ESM.docx]

***Online repository***

**Methods**

***Measurements and calculations***

*Height estimations formulas*

They were performed using a 300 mm and a 800 mm precision metal callipers. Seven different limb measurements (Figure 1) were used to determinate height estimation (*Calculated height*) using 18 different anthropometric formulas during the preliminary phase, on healthy volunteers.

Index distal phalange

Male Height I1 (cm) = 151.88 + [9.79 x Lenght (cm)] Female Height I1 (cm) = 145.78 + [6.69 x Lenght (cm)]

Male Height I2 (cm) = 152.38 + [9.79 x Lenght (cm)] Female Height I2 (cm) = 148.78 + [6.69 x Lenght (cm)]

Hand Lenght

Male Height HL1 (cm) = 94.835 + [4.187 x Lenght (cm)] Female Height HL1 (cm) = 74.404 + [4.945 x Lenght (cm)]

Male Height HL2 (cm) = 88.243 + [4.39 x Lenght (cm)] Female Height HL2 (cm) = 81.314 + [4.42 x Lenght (cm)]

Male Height HL3 (cm) = 55.7 + [6.06 x Lenght (cm)] Female Height HL3 (cm) = 101.13 + [3.39 x Lenght (cm)]

Hand Width

Male and Female Height HW1 (cm) = 66.366 + [4.031 x Lenght (cm)] + [3.177 x Width (cm)] (16)

Male Height HW2 (cm) = 98.23 + [8.51 x Lenght (cm)] Female Height HW2 (cm) = 110.39 + [6.13 x Lenght (cm)]

**Ulna**

Male Height U1 (cm) = 74.15 + [3.7 x Lenght (cm)] Female Height U1 (cm) = 57.76 + [4.27 x Lenght (cm)]

Male Height U2 (cm) = 76.65 + [3.7 x Lenght (cm)] Female Height U2 (cm) = 60.26 + [4.27 x Lenght (cm)]

Male Height U3 (cm) = 34.95 + [5.17 x Lenght (cm)] Female Height U3 (cm) = 25.53 + [5.63 x Lenght (cm)]

**Tibia**

Male and Female Height T1 (cm) = 91.19 + [2 x Lenght (cm)] (20)

Male Height T2 (cm) = 70.18 + [2.552 x Lenght (cm)] Female Height T2 (cm) = 61.89 + [2.7 x Lenght (cm)]

Male Height T3 (cm) = 78.52 + [2.52 x Lenght (cm)] Female Height T3 (cm) = 61.53 + [2.9 x Lenght (cm)]

Male Height T4 (cm) = 81.02 + [2.52 x Lenght (cm)] Female Height T4 (cm) = 64.03 + [2.9 x Lenght (cm)]

Male Height T5 (cm) = 53.36 + [3.18 x Lenght (cm)] Female Height T5 (cm) = 42 + [3.44 x Lenght (cm)]

**Chumlea**

Male Height (cm) = 64.19 - [0.04 x age (years)] + [2.03 x Lenght (cm)]

Female Height (cm) = 84.88 - [0.24 x age (years)] + [1.83 x Lenght (cm)]

*Ideal and predicted body weight estimations*

IBW

Male IBW (kg) = Height (cm) – 100 – (Height – 150)/4

Female IBW (kg) = Height (cm) – 100 – (Height – 150)/2.5

PBW

Males PBW (kg) = 50 + 0.91 [Height (cm) – 152.4].

Females: PBW (kg) = 45.5 + 0.91 [Height (cm) – 152.4]
